# Metabolic and microstructural brain alterations associated with occupational blast exposure

**DOI:** 10.1016/j.ynirp.2026.100416

**Published:** 2026-09-29

**Authors:** Chihwa Song, Chandler Sours Rhodes, Sara M. Lippa, Ping-Hong Yeh, Jacob R. Powell, Treven C. Pickett

**Affiliations:** a National Intrepid Center of Excellence, Walter Reed National Military Medical Center, Bethesda, MD, 20889, USA; b Department of Medical and Clinical Psychology, Uniformed Services University of the Health Sciences, Bethesda, MD, 20889, USA; c Department of Diagnostic Radiology and Nuclear Medicine, University of Maryland Medical School, Baltimore, MD, 21201, USA; d Department of Neuroscience, Uniformed Services University of the Health Sciences, Bethesda, MD, 20889, USA; e Department of Physical Medicine and Rehabilitation, Uniformed Services University of the Health Sciences, Bethesda, MD, 20814, USA

**Keywords:** FDG PET, MOS, Blast, Pallidal lateralization, Traumatic brain injury, Military

## Abstract

Repeated occupational exposure to low-level blast may pose a risk to the neurological health of service members. To date, no studies have specifically investigated the effects of such repetitive exposure using fluorodeoxyglucose positron emission tomography (FDG-PET). This study aims to characterize cerebral metabolic alterations associated with occupational blast exposure, accounting for confounding factors such as posttraumatic stress disorder (PTSD) symptoms and aging. Male service members with chronic mild TBI (mTBI) were categorized into high-risk (HR; n = 204) and low-risk (LR; n = 68) blast exposure groups based on their military occupational specialty. FDG-PET and diffusion tensor imaging (DTI) data were analyzed using region-of-interest (ROI) and voxelwise methods. Statistical models included age and PTSD symptom severity as covariates. Compared to the LR group, ROI analyses revealed higher FDG uptake in the left pallidum, caudate, and ventrolateral prefrontal cortex in the HR group. These elevations in metabolic activity may reflect compensatory mechanisms in response to neurophysiological disruption following blast exposure. Voxelwise analysis showed lower FDG uptake in the HR group within white matter, predominantly in the right hemisphere, including the corpus callosum, anterior thalamic radiation, inferior and superior longitudinal fasciculi. Corresponding DTI findings showed higher fractional anisotropy (p = 0.0025) and axial diffusivity (p = 0.024) in these regions. Furthermore, leftward metabolic lateralization was observed in the pallidum, with the HR group (Cohen's d = 1.36, 95% CI [1.14, 1.57]) exhibiting greater metabolic asymmetry than the LR group (Cohen's d = 1.16, 95% CI [0.79, 1.51]). Findings from this study reveal metabolic and microstructural alterations associated with occupational blast exposure in service members with mTBI.

## 1 Introduction

Repeated exposure to low-level blast is a common occurrence in military and law enforcement settings, arising from routine training and operational activities. Although the nature of blast exposure such as its magnitude, frequency, and periodicity varies by occupational role, concerns have been raised regarding its potential impact on brain health. While existing evidence indicates that low-level blast may pose risks to affected populations, the findings remain inconclusive, highlighting the need for further research (Belding et al., 2021a).

Cumulative neurological alterations that may develop in service members over a career of repeated low-level blast exposures have been examined using neuroimaging techniques (Stone et al., 2020; Yuan et al., 2019). However, no studies have specifically investigated the effects of repeated occupational exposure to low-level blast using fluorodeoxyglucose positron emission tomography (FDG-PET). Unlike blood-oxygen-level-dependent functional MRI (fMRI), which reflects hemodynamic responses, FDG-PET measures regional cerebral glucose metabolism and may identify persistent metabolic alterations not readily captured by fMRI. While previous research has explored cerebral metabolic changes following high-level blast exposure in traumatic brain injury (TBI), the findings have been inconsistent. For instance, one study found that a higher number of prior blast exposures was associated with lower cerebellar metabolism in 33 military veterans (Meabon et al., 2016). Another study reported hypometabolism in the cerebellum, vermis, pons, and medial temporal lobe in 12 blast-exposed veterans with chronic post-concussive symptoms, compared to 12 controls (Peskind et al., 2011). In contrast, a more recent study reported significantly increased FDG uptake in the left pallidum of 79 combat veterans with retrospectively recalled blast-related mild TBI (mTBI), compared to 41 control participants, and proposed it as an imaging biomarker for blast mTBI (Terry et al., 2024).

Moreover, blast exposure and post-traumatic stress symptoms often coexist, especially in military populations. FDG-PET studies in post-traumatic stress disorder (PTSD) have shown altered cerebral glucose metabolism. Our previous study reported altered metabolic interrelationships in the cortico-limbic network in service members with mTBI and persistent PTSD symptoms compared to those with mTBI but without PTSD symptoms (Song et al., 2022). Another study found that participants with PTSD exhibited increased stress-associated neural network activity, reflected in greater amygdala activity relative to ventromedial prefrontal cortex activity, compared to controls (Gharios et al., 2024). It is possible that pre-existing differences in metabolism may represent a risk factor for PTSD, as one study revealed greater regional cerebral metabolic rates of glucose (rCMRglu) in the temporal, occipital, fusiform cortices and cerebellum, and lower rCMRglu in the insula, cingulate gyri, and hippocampus (Molina et al., 2010). Moreover, veterans with comorbid mTBI and PTSD had larger clusters of low uptake voxels than healthy veterans (Buchsbaum et al., 2015). These findings underscore the importance of accounting for PTSD when investigating the effects of blast exposure on cerebral glucose metabolism.

Because white matter tracts are known to be particularly vulnerable to blast-related shear forces, we additionally examined diffusion tensor imaging (DTI) metrics within white matter regions showing metabolic group differences to assess corresponding microstructural differences. This study aimed to compare cerebral metabolism and white matter microstructure between cohorts at high and low occupational risk for blast exposure. We hypothesized that service members at high risk for repetitive blast exposure would exhibit cerebral metabolic and microstructural alterations compared to those at low risk. To evaluate the effects of occupational blast exposure, we employed both region-of-interest (ROI) and voxelwise analyses to assess the spatial distribution of altered FDG uptake. Given the potential influence of age and PTSD symptom severity on FDG uptake, these variables were included as covariates in our statistical models.

## 2 Materials and methods

### 2.1 Participants

A total of 272 male service members with mTBI were included in this study. This study was approved by the Institutional Review Board at the Walter Reed National Military Medical Center (WRNMMC) and is compliant with the Health Insurance Portability and Accountability Act guidelines. Active-duty military service members enrolled in the four-week Intensive Outpatient Program (IOP) at the National Intrepid Center of Excellence (NICoE) provided informed consent. Service members were referred to the NICoE IOP for treatment of TBI and/or comorbid psychological health symptoms. Participants with a history of moderate to severe TBI or major neurologic or psychiatric conditions such as psychosis, stroke, multiple sclerosis, or spinal cord injury were excluded. FDG-PET and MRI were acquired in the second week of the four-week program.

### 2.2 Clinical measures

Study participants completed the PTSD Check List - Civilian Version (PCL-C) (Blanchard et al., 1996) and Neurobehavioral Symptom Inventory (NSI) (Cicerone and Kalmar, 1995). The PCL-C is a standardized self-report rating scale comprising 17 items that correspond to the key symptoms of PTSD. These 17 items are summed to calculate the total score, which was used to represent PTSD symptom burden. NSI is a measure of post-concussive symptoms. Research on the NSI has resulted in the Validity-10 scale, a screener for potential symptom exaggeration. Participants with a Validity-10 score greater than 25 were excluded (n = 21; Dretsch et al., 2017).

### 2.3 MOS categorization

For each participant, self-reported military occupational specialty (MOS) was collected from intake paperwork collected upon admission to the NICoE IOP. Participants were classified into high-risk (HR) and low-risk (LR) occupational blast exposure groups using the MOS categorization developed by Belding and coworkers (Belding et al., 2021b). To improve accuracy of group categorization, available MOS data were cross-referenced with participants’ military service records. Due to the data type being an open text field, only MOS data that could be clearly identified either by classification code (i.e., 11B) or text description (i.e., infantry) were included in this categorization. The HR and LR groups were matched for age and PCL-C total score. Because both groups were enrolled in the same standard IOP curriculum, the early educational and intervention components prior to imaging were expected to be comparable between groups and therefore unlikely to confound group differences.

### 2.4 Image acquisition

All study participants were scanned with a PET/CT scanner (Biograph 64 mCT, Siemens Healthcare, Knoxville, TN). A static PET/CT scan was performed at 50 min after intravenous injection of ^18^F-FDG (9 - 11 mCi). Participants fasted for 6 - 8 h prior to the scan. The 20-min PET images were reconstructed using the ordered subset expectation maximization (OSEM) algorithm with 6 iterations and 24 subsets (matrix = 400 × 400 × 148, voxel size = 1 × 1 × 1.5 mm^3^). Additionally, participants were scanned with a 3T MRI scanner (Discovery MR 750, GE Healthcare, Milwaukee, WI) using a 32-channel head coil (MR Instruments, Minneapolis, MN). T1-weighted MR images were acquired with a 3D BRAVO sequence (TR/TE = 6.7/2.5 ms, FA = 12^o^, matrix = 512 x 512 x 312, voxel size = 0.5 × 0.5 × 0.6 mm^3^). Additionally, diffusion-weighted imaging (DWI) was performed using a single shot dual spin-echo echo planar imaging sequence with slice-selection gradient reversal and peripheral cardiac-gating (TR/TE = 10,000/90 ms, matrix = 128 × 128 × 70, voxel size 2 × 2 × 2 mm^3^, b = 1000 s/mm^2^). A total of 48 non-collinear diffusion gradient directions were acquired, along with 7 non-diffusion weighted (b = 0) volumes.

### 2.5 Image processing

FDG-PET images were normalized to the mean activity of the whole brain and smoothed using a 4 mm full-width at half-maximum (FWHM) Gaussian kernel. T1-weighted MR images were processed with FreeSurfer 5.3 (Fischl, 2012) to segment subcortical structures and cortical regions according to the Desikan–Killiany atlas. Prefrontal cortex (PFC) subregions were divided into the dorsolateral PFC (dlPFC), ventrolateral PFC (vlPFC), and ventromedial PFC (vmPFC), and the remaining cortical regions was grouped by lobe. FDG-PET images were registered to corresponding T1 scans, and mean FDG uptake values were calculated across 39 brain regions. For voxelwise analysis, PET images were spatially normalized to the MNI152 standard space. Preprocessing of DWI data included correction of EPI geometric distortion using B0 fieldmap, correction of motion and eddy current artifacts, and brain extraction using the FSL toolkit (Jenkinson et al., 2012). DTI scalar maps were generated using logarithmic linear regression for fractional anisotropy (FA), mean diffusivity (MD), axial diffusivity (AD), and radial diffusivity (RD).

### 2.6 Statistical analyses

Group differences in age, PCL-C and NSI total scores, and years of education were assessed using either two-sample t-tests or Mann-Whitney U tests, based on the data's distribution. The relationship between FDG uptake and MOS risk group was investigated in 39 brain regions using generalized linear models with a gamma distribution and an identity link function, controlling for age and PCL-C total score. Normalized FDG uptake values were scaled by a factor of 100. To evaluate the model's fit, the ratio of the scaled deviance (D*) to the degrees of freedom (df) was calculated. A ratio close to 1 suggests that the gamma distribution provides an adequate fit to the data. To control for multiple comparisons, a false discovery rate (FDR) correction was applied with a threshold of q = 0.05. Variance inflation factors (VIFs) were calculated to assess multicollinearity among predictors, with values below 5 considered acceptable. A significance level of p < 0.05 was used for all statistical tests. All analyses were conducted using R 4.2.3 (R Core Team, 2021).

For the voxelwise analysis, a non-parametric permutation test was performed using FSL's *randomise* tool (Jenkinson et al., 2012), with age and PCL-C total score included as covariates. Threshold-Free Cluster Enhancement (TFCE) was applied to detect clusters exhibiting significant group differences. Statistical significance was determined using family-wise error (FWE) correction at pFWE < 0.05 across the whole brain, based on 10,000 permutations. Mean DTI metrics were calculated across all voxels exhibiting statistically significant group differences in FDG uptake.

In sensitivity analyses, the generalized linear models for all 39 regions were rerun with NSI total score included as an additional covariate to assess whether the associations between MOS risk group and regional FDG uptake were robust to adjustment for post-concussive symptom burden.

## 3 Results

### 3.1 Participant characteristics

A total of 272 male participants were analyzed in the final dataset. Participant characteristics are summarized in Table 1. No significant differences were observed between the HR (n = 204) and LR (n = 68) groups for age and PCL-C total score. However, the HR group reported a greater number of years of education (p = 0.004) and significantly lower NSI total score (p = 0.004).

**Table 1:** Participant characteristics.

	High Risk (n = 204)	Low Risk (n = 68)	p
Age (years)	35.3 ± 6.5	33.6 ± 7.7	0.069
Education (years)	14.2 ± 1.9	13.5 ± 2.0	0.004
Race (%)			
Asian	3.9	5.9	
Black	1.5	2.9	
Indian	1.5	0.0	
White	89.7	80.9	
Unknown	3.4	10.3	
Branch			
Army	21.1	48.5	
Air Force	9.3	11.8	
Military Academy	0.5	0.0	
Marine Corp	14.2	27.9	
Navy	53.4	11.8	
National Guard	1.5	0.0	
PCL-C total	50.2 ± 13.8	54.1 ± 16.2	0.078
NSI total	35.7 ± 13.6	41.6 ± 13.0	0.004

Note: Mean ± standard deviation.

### 3.2 Regional group comparisons

All regional analyses were adjusted for age and PCL-C total score. Generalized linear regression analyses of the 39 brain regions revealed significantly greater FDG uptake in the HR group, compared to the LR group, in the left pallidum, caudate and vlPFC after correction for multiple comparisons (summarized in Table 2). For all ROIs, the ratio of scaled deviance to degrees of freedom (D*/df) was approximately 1, indicating a good model fit. The highest VIF across all models was 1.03, suggesting minimal multicollinearity. After FDR correction, FDG uptake was significantly associated with age in several subcortical and cortical regions, as well as brainstem (Table 2). In contrast, PCL-C total score was associated with FDG uptake in the bilateral parietal cortices; however, this association did not survive FDR correction.

**Table 2:** Coefficients, p-values, q-values, and ratios of scaled deviance to degrees of freedom (D*/df) from multivariable generalized linear models of regional FDG uptake simultaneously including MOS risk group, PCL-C total score, and age as predictors.

ROI	Risk			PCL-C total			Age			D*/df
	b	p-value	q-value	b	p-value	q-value	b	p-value	q-value	
Thalamus (L)	1.655	0.068	0.177	−0.009	0.752	0.822	0.314	**0.000**	**0.000**	0.990
Thalamus (R)	2.482	**0.012**	0.079	−0.029	0.319	0.708	0.307	**0.000**	**0.000**	0.988
Caudate (L)	3.357	**0.003**	**0.047**	−0.056	0.096	0.578	−0.024	0.732	0.845	1.020
Caudate (R)	2.481	**0.017**	0.083	−0.039	0.207	0.578	−0.020	0.766	0.845	1.006
Putamen (L)	2.226	**0.020**	0.083	−0.039	0.176	0.578	0.145	**0.017**	**0.035**	0.997
Putamen (R)	2.530	**0.010**	0.079	−0.053	0.071	0.578	0.242	**0.000**	**0.001**	0.995
Pallidum (L)	4.751	**0.001**	**0.047**	0.019	0.674	0.821	0.349	**0.000**	**0.001**	0.973
Pallidum (R)	2.081	**0.047**	0.142	−0.043	0.172	0.578	0.298	**0.000**	**0.000**	0.969
Hippocampus (L)	−0.091	0.881	0.922	−0.016	0.394	0.767	0.082	**0.035**	0.066	1.004
Hippocampus (R)	−0.239	0.686	0.882	−0.023	0.194	0.578	0.134	**0.000**	**0.001**	1.004
Amygdala (L)	−0.225	0.724	0.882	−0.017	0.375	0.767	0.111	**0.006**	**0.015**	1.000
Amygdala (R)	−0.146	0.819	0.922	−0.014	0.466	0.809	0.124	**0.002**	**0.006**	1.003
Accumbens (L)	1.651	0.141	0.306	−0.015	0.650	0.821	0.392	**0.000**	**0.000**	1.013
Accumbens (R)	−0.119	0.900	0.922	−0.005	0.849	0.849	0.370	**0.000**	**0.000**	0.995
VentralDC (L)	0.432	0.453	0.664	−0.009	0.602	0.809	0.235	**0.000**	**0.000**	0.999
VentralDC (R)	0.486	0.405	0.660	−0.012	0.487	0.809	0.232	**0.000**	**0.000**	1.000
dlPFC (L)	1.786	**0.012**	0.079	0.033	0.125	0.578	−0.061	0.179	0.279	0.998
dlPFC (R)	1.240	0.074	0.179	0.034	0.099	0.578	−0.019	0.659	0.803	0.999
vlPFC (L)	2.345	**0.004**	**0.047**	0.013	0.580	0.809	0.007	0.894	0.943	0.994
vlPFC (R)	1.701	**0.027**	0.094	0.005	0.818	0.839	0.034	0.487	0.679	0.992
vmPFC (L)	1.674	**0.021**	0.083	0.014	0.519	0.809	−0.003	0.951	0.969	1.007
vmPFC (R)	1.029	0.164	0.337	0.014	0.528	0.809	−0.025	0.591	0.769	1.008
Frontal cortex (L)	1.363	**0.019**	0.083	−0.020	0.257	0.625	−0.039	0.294	0.424	0.995
Frontal cortex (R)	1.131	0.067	0.177	−0.008	0.655	0.821	0.011	0.780	0.845	0.995
Temporal cortex (L)	−0.339	0.526	0.732	0.018	0.255	0.625	−0.001	0.969	0.969	0.994
Temporal cortex (R)	−0.439	0.407	0.660	0.020	0.196	0.578	0.037	0.265	0.398	0.990
Parietal cortex (L)	0.809	0.179	0.350	0.042	**0.020**	0.578	0.074	0.054	0.095	0.997
Parietal cortex (R)	0.261	0.648	0.871	0.036	**0.036**	0.578	0.093	**0.011**	**0.025**	0.994
Occipital cortex (L)	−0.695	0.423	0.660	0.014	0.586	0.809	0.096	0.081	0.138	1.013
Occipital cortex (R)	−0.621	0.459	0.664	−0.008	0.759	0.822	0.117	**0.028**	0.054	1.000
ACC (L)	0.300	0.715	0.882	0.036	0.138	0.578	−0.083	0.111	0.180	0.999
ACC (R)	0.087	0.922	0.922	0.026	0.327	0.708	−0.019	0.742	0.845	1.010
PCC (L)	1.386	0.099	0.226	0.008	0.759	0.822	0.132	**0.014**	**0.029**	0.997
PCC (R)	1.643	**0.045**	0.142	0.017	0.486	0.809	0.201	**0.000**	**0.001**	0.999
Insula (L)	0.534	0.392	0.660	−0.026	0.163	0.578	−0.019	0.633	0.796	0.998
Insula (R)	0.504	0.419	0.660	−0.032	0.083	0.578	−0.021	0.591	0.769	0.997
Cerebellar cortex (L)	−0.134	0.872	0.922	0.006	0.798	0.839	0.299	**0.000**	**0.000**	1.007
Cerebellar cortex (R)	−0.169	0.847	0.922	−0.008	0.759	0.822	0.279	**0.000**	**0.000**	1.006
Brainstem	0.070	0.909	0.922	−0.010	0.598	0.809	0.202	**0.000**	**0.000**	1.006

Note: Significant p-values and q-values are presented in bold.

Note: ACC = anterior cingulate cortex; DC = diencephalon; dlPFC = dorsolateral prefrontal cortex (PFC); PCC = posterior cingulate cortex; vlPFC = ventrolateral PFC; vmPFC = ventromedial PFC.

In sensitivity analyses that included NSI total score as an additional covariate, NSI itself was not significantly associated with FDG uptake in any of the 39 regions (all uncorrected p > 0.05). The original group differences in the left pallidum, caudate, and vlPFC did not retain statistical significance after FDR correction (q = 0.066–0.077).

### 3.3 Voxelwise comparisons

Voxelwise analysis did not reveal significant group differences in FDG uptake in the gray matter regions. However, lower FDG uptake was observed in the HR group within white matter, predominantly in the right hemisphere, including the corpus callosum, anterior thalamic radiation, and inferior and superior longitudinal fasciculi, compared to the LR group (Fig. 1). Additionally, smaller clusters were observed in the left hemisphere. These clusters survived whole-brain family-wise error correction (pFWE < 0.05). Within these regions, the HR group had higher FA (p = 0.0025) and AD (p = 0.024), but no significant differences were found for MD or RD. Adjusted means with 95% confidence intervals, controlling for age and PCL-C total score, are presented for each group in Fig. 2.

**Fig. 1:**
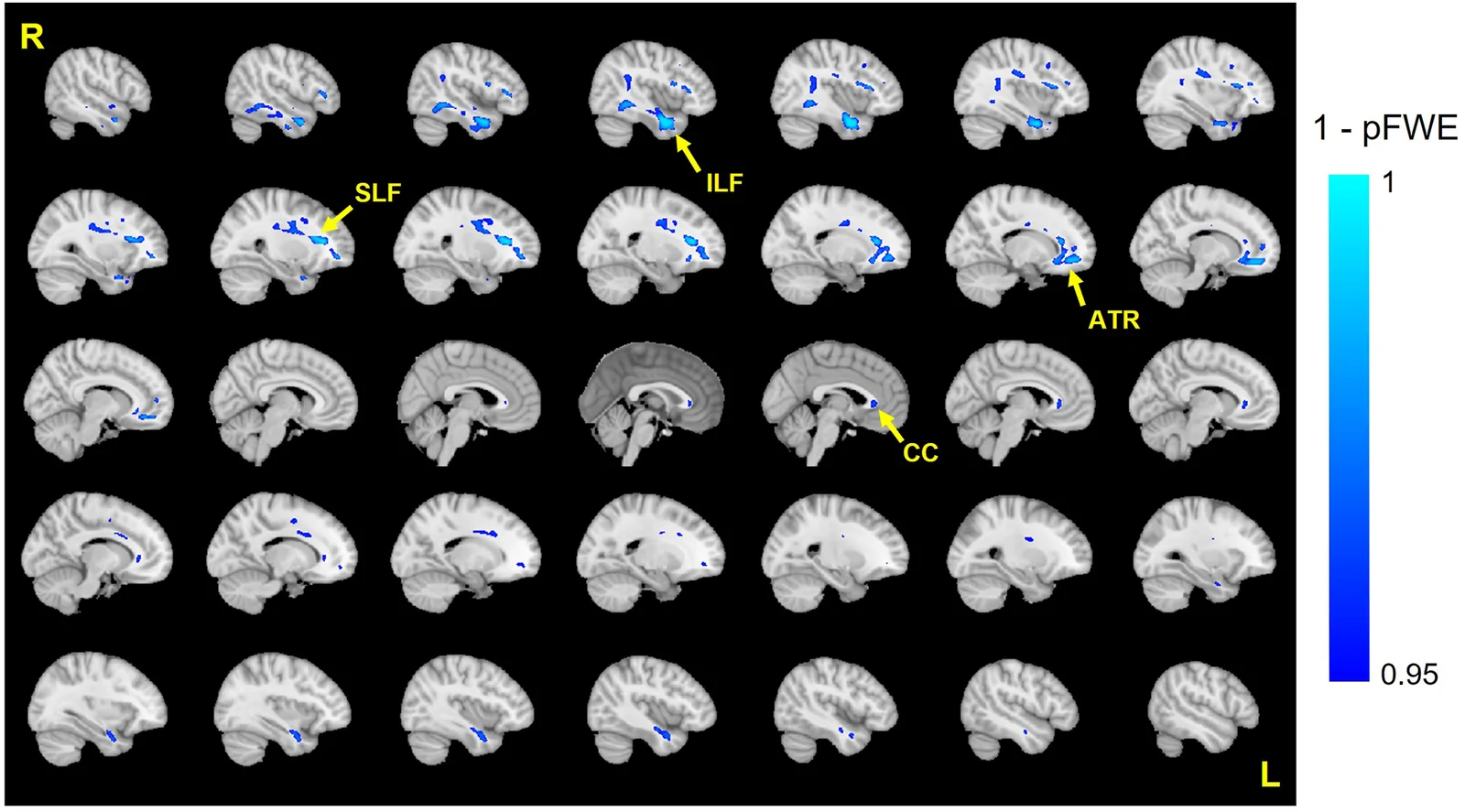
FWE-corrected p-value (pFWE) maps of voxelwise group differences. A non-parametric permutation test was performed with age and PCL-C total score as covariates. Compared to the LR group, the HR group showed significantly reduced FDG uptake in the corpus callosum (CC), anterior thalamic radiation (ATR), and inferior and superior longitudinal fasciculi (ILF and SLF), predominantly in the right hemisphere. Additionally, smaller clusters were observed in the left hemisphere. No significant differences were observed in cortical or subcortical regions.

**Fig. 2:**
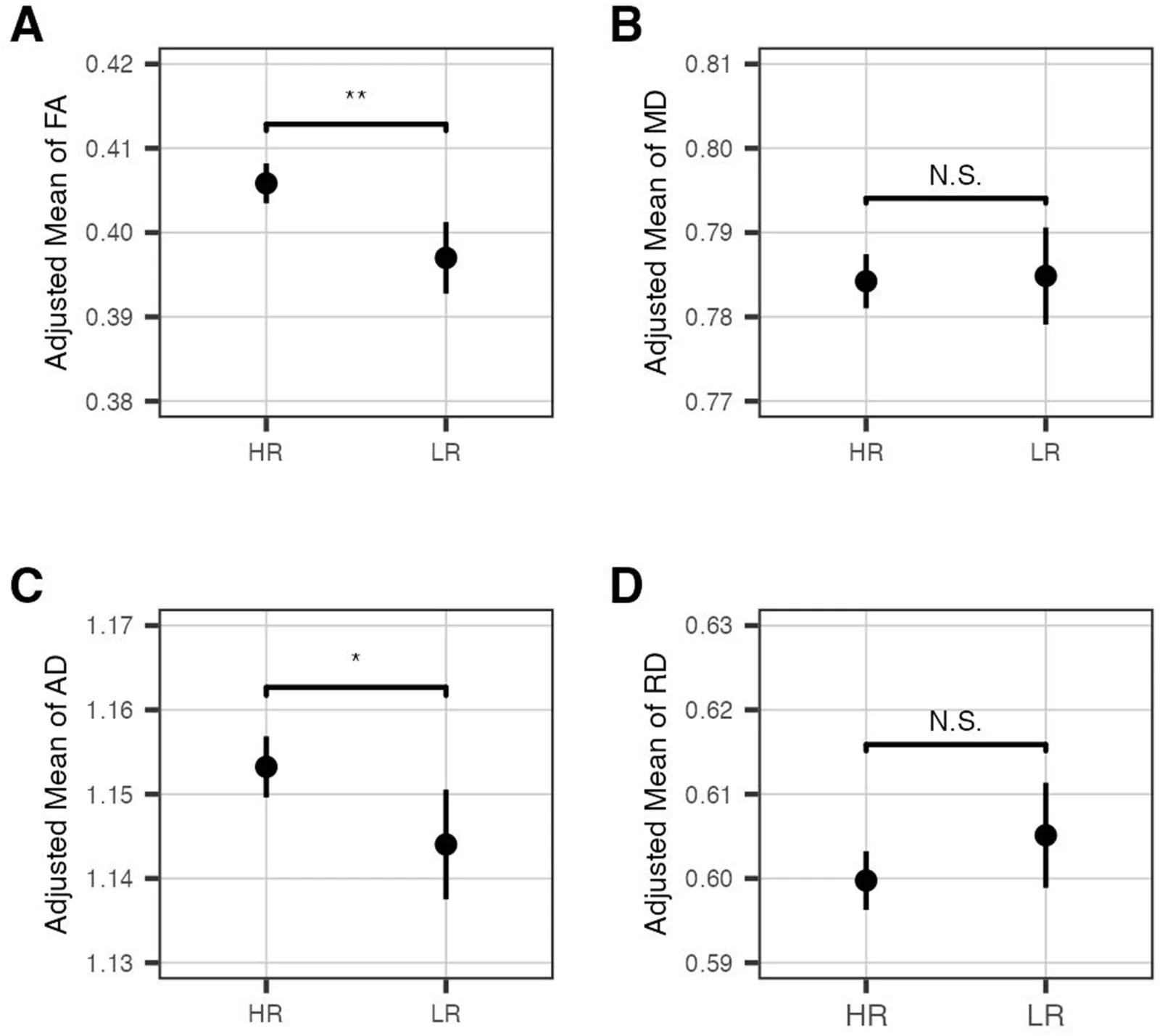
Group comparisons of DTI metrics in the regions exhibiting significant metabolic differences. Adjusted means with 95% confidence intervals, controlling for covariates, are presented for each group. The HR group showed higher FA (A) and AD (C) values; however, no statistically significant differences were observed between groups in MD (B) or RD ().

### 3.4 Lateralization of FDG uptake

Since the ROI analyses revealed significant group differences only in left hemisphere regions, additional analyses were conducted to assess left-right asymmetry in FDG uptake. The effect size for FDG uptake between the left and right vlPFC was small in both the HR (Cohen's d = −0.13, 95% CI [−0.33, 0.06]) and LR (Cohen's d = −0.26, 95% CI [−0.60, −0.08]) groups. However, pronounced leftward lateralization was observed in the pallidum, with the HR group showing greater metabolic asymmetry (Cohen's d = 1.36, 95% CI [1.14, 1.57]) compared to the LR group (Cohen's d = 1.16, 95% CI [0.79, 1.51]). In contrast, rightward lateralization was found in the caudate, where the HR group showed reduced metabolic asymmetry (Cohen's d = −0.48, 95% CI [−0.68, −0.29]) relative to the LR group (Cohen's d = −0.60, 95% CI [−0.95, −0.26]).

We also examined volumetric asymmetry in the pallidum, which exhibited significant metabolic asymmetry. The LR group showed a moderate left-right asymmetry (Cohen's d = 0.41, 95% CI [0.07, 0.75]), which is comparable to the value reported for healthy controls in the ENIGMA (Enhancing Neuro-Imaging Genetics through Meta-Analysis) study (Kong et al., 2022). In contrast, the HR group exhibited greater volumetric asymmetry (Cohen's d = 0.81, 95% CI [0.61, 1.01]). No significant differences in pallidal volume asymmetry were observed between left- and right-handed individuals (t = −0.41, p = 0.68).

## 4 Discussion

Among the 39 cortical and subcortical regions analyzed, the HR group exhibited greater FDG uptake in the left pallidum, caudate, and vlPFC, compared to the LR group. The increased uptake in the left pallidum aligns with a recent study of 79 combat veterans (Terry et al., 2024), and contrasts with earlier studies with smaller sample sizes that reported no overlapping regions. Additionally, regression analyses indicated that FDG uptake was significantly associated with age in several cortical and subcortical regions, but not with PTSD symptom severity. Furthermore, voxelwise analysis revealed that the HR group had lower FDG uptake within white matter, with these regions exhibiting higher fractional anisotropy (FA) and axial diffusivity (AD).

The multimodal pattern may reflect two complementary processes: concurrent metabolic and microstructural alterations in vulnerable white matter tracts and compensatory hypermetabolism in key gray matter nodes of the cortico-basal-ganglia circuitry. Elevated FDG uptake in left pallidum, caudate, and vlPFC may reflect compensatory upregulation. Greater leftward metabolic and volumetric asymmetry of the pallidum further supports a lateralized compensatory process. In parallel, the convergence of reduced FDG uptake and altered diffusion metrics in these white matter regions may indicate concurrent metabolic and microstructural alterations associated with occupational blast-exposure risk.

### 4.1 Increased FDG uptake in cortical and subcortical regions

The observed increase in FDG uptake in the left pallidum, caudate, and vlPFC may reflect a compensatory response to neurophysiological disruption following blast exposure. The vlPFC and basal ganglia are functionally interconnected via cortico-basal ganglia circuitry, which plays essential roles in motor control, goal-directed behavior, reward-based learning, and habit formation (Mink, 1996; Balleine and O'Doherty, 2010; Ochsner et al., 2012; Buhle et al., 2014; Haber, 2016). The basal ganglia are capable of undergoing neuroplastic changes to preserve function following injury (Graybiel, 2004; Mink, 2018; Roth and Ding, 2024), and compensatory processes may also involve the recruitment of alternative brain regions not associated with the affected area (Bezard et al., 2001; Appel-Cresswell et al., 2010). In Parkinson's disease, such compensatory mechanisms are believed to delay or attenuate the onset and severity of motor symptoms (Bezard and Gross, 1998; Brotchie and Fitzer-Attas, 2009; Alshahrani et al., 2025), although evidence suggests that compensation may also contribute to disease progression in some cases (Brotchie and Fitzer-Attas, 2009; Blesa et al., 2017).

Inconsistent findings in previous FDG-PET studies may be attributed to small sample sizes and participant heterogeneity in demographics and exposure histories. Additionally, variability in results may stem from differences in blast exposure characteristics, such as force intensity, direction, co-occurring injury mechanisms, and individual protective or resilience factors. For example, a simulation study using fluid-structure interaction modeling demonstrated that blast orientation significantly affects injury distribution, with lateral blasts producing more diffuse stress fields across the brain and frontal blasts resulting in localized damage to anterior cortical regions (Amponsah et al., 2025). In the current study, ROI analyses revealed significant differences in FDG uptake in cortical and subcortical regions, whereas voxelwise comparisons did not reach significance at the given threshold. This suggests that metabolic alterations may be spatially localized but lack consistent spatial patterns within regions across individuals.

Our understanding of the precise mechanisms by which blast waves damage the brain is still developing. Studies suggest that blast TBI is primarily an interface-driven injury, where mechanical forces concentrate at anatomical boundaries including gray-white matter junctions, cortical sulci, cerebrospinal fluid spaces, and perivascular structures (Miller et al., 2021; Su and Bell, 2016). However, such localized effects were not observed in the current study, likely because the limited spatial resolution of PET imaging lacks the sensitivity to detect microstructural damage at these interfaces.

### 4.2 Lateralization of pallidal metabolism

In our study, no significant metabolic alteration was observed in the right pallidum after FDR correction. In contrast, Terry et al. reported increased FDG uptake in the right pallidum, though the effect was less pronounced than in the left, resulting in leftward lateralization in that region (Terry et al., 2024). Similarly, our findings demonstrated leftward lateralization in the pallidum, with the HR group showing greater metabolic asymmetry compared to the LR group. A similar pattern emerged in pallidal volume, with leftward lateralization and greater volumetric asymmetry in the HR group compared to the LR group. These findings suggest that lateralized alterations in pallidal FDG uptake and volume may serve as imaging biomarkers for repeated low-level blast exposure.

### 4.3 White matter hypometabolism

Unlike cortical and subcortical regions, voxelwise analysis reveals hypometabolism within white matter including the corpus callosum, anterior thalamic radiation, and inferior and superior longitudinal fasciculi. White matter is well established as particularly vulnerable to injury following TBI (Hulkower et al., 2013). The observed hypometabolism in white matter can be explained by reactive gliosis, also known as glial scar formation, leading to cellular hypertrophy and the creation of a protective but potentially inhibitory scar (Gao et al., 2013). While reactive gliosis serves to contain damage and provide initial support, chronic neuro-inflammation can hinder axonal regrowth and contribute to secondary degeneration and neurodegenerative disease progression through excessive activation of glial cells that release pro-inflammatory cytokines and chemokines (Adamu et al., 2024; Huang et al., 2025; Smith et al., 2012; Zhang et al., 2023). Glial scar formation acts as a significant physical and chemical barrier to neuronal regeneration by establishing a dense, isolating matrix that creates an inhibitory environment (Cregg et al., 2014; Wang et al., 2018). This can result in neuronal dysfunction or death, and the inability of neurons to form new connections or repair existing ones leads to reduced neuronal activity and, consequently, decreased metabolic demand, creating a localized area of hypometabolism.

### 4.4 Microstructural changes in white matter

If white matter tracts were damaged in a typical manner such as through demyelination or axonal degeneration, FA would generally be expected to decrease, reflecting a loss of structural integrity and directional coherence in the tissue. However, in contrast to this expectation, the current study observed higher FA and AD in the HR group within white matter regions showing hypometabolism. This seemingly paradoxical finding may be explained by damage to crossing white matter fibers. When one set of crossing fibers is selectively disrupted, the remaining intact fibers can dominate the diffusion signal, thereby leading to an apparent increase in FA. Therefore, our observation of elevated FA may reflect a specific pattern of microstructural damage rather than enhanced white matter integrity.

DTI studies on low-level blast exposure have been mixed, with some reported no alterations in DTI metrics in cases of low-level blast (Champagne et al., 2021; Lippa et al., 2023; Meabon et al., 2016; Stone et al., 2024), while others found reductions in FA (or relative anisotropy) in white matter regions (Davenport et al., 2012; Ivanov et al., 2017; Stone et al., 2020). These discrepancies may be attributed to methodological differences, including evaluation of blast exposure, correction for multiple comparisons, and sample sizes, some of which are notably small.

### 4.5 Limitations

We classified participants into blast risk groups based on their MOS. However, MOS-based classification does not account for the wide variability in individual exposure such as the blast's magnitude, frequency, or the duration of each event. Additionally, an individual's MOS can change over the course of their military career. Future studies would benefit from incorporating self-report measures such as the Blast Exposure Threshold Survey (BETS) (Modica et al., 2021) and the NICoE Blast Ordnance and Occupational Exposure Measure (BOOM) (Sours Rhodes et al., 2023) to quantify career blast exposure history in a consistent manner. Another important limitation is the absence of a non-exposed control group. The lack of a control group limits the ability to distinguish blast-related effects from other potential confounders such as PTSD, mTBI, or other military occupational exposures. Including a well-matched control group in future research will be critical for isolating the specific contributions of blast exposure to observed brain changes. Additionally, prior studies have reported age-related variation in cerebral metabolism (Qian et al., 2025). However, without a non-mTBI, non-blast-exposed control group, we cannot determine whether these age associations reflect typical aging, injury-related effects, blast-related effects, or a combination of these factors. Lastly, a key limitation of DTI is its inability to resolve multiple fiber orientations within a single voxel, which is a problem as up to 90% of white matter voxels contain crossing fibers (Jeurissen et al., 2013). Higher-order diffusion MRI samples water diffusion with greater detail by using a larger number of diffusion-sensitizing gradients. By capturing non-Gaussian water diffusion behavior, these methods can infer more complex tissue structures, enabling more accurate assessments of axonal integrity and damage.

## 5 Conclusion

This study found that high blast-exposure risk is associated with increased FDG uptake in the left pallidum, caudate, and vlPFC, which may reflect compensatory mechanisms. Additionally, greater leftward pallidal lateralization and white matter hypometabolism were observed, with increased FA and AD indicating selective disruption of crossing fibers. FDG uptake correlated with age in certain brain regions but showed no association with PTSD symptom severity. Our findings reveal distinct metabolic and microstructural alterations in both gray and white matter of service members at high risk of occupational blast exposure.

## CRediT authorship contribution statement

**Chihwa Song:** Writing – review & editing, Writing – original draft, Methodology, Formal analysis, Conceptualization. **Chandler Sours Rhodes:** Writing – review & editing, Data curation, Conceptualization. **Sara M. Lippa:** Writing – review & editing, Conceptualization. **Ping-Hong Yeh:** Writing – review & editing, Conceptualization. **Jacob R. Powell:** Writing – review & editing, Conceptualization. **Treven C. Pickett:** Writing – review & editing, Conceptualization.

## Disclaimer

The views expressed in this manuscript are those of the authors and do not necessarily reflect the official policy of the Department of War or the US Government.

## Declaration of competing interest

The authors declare that they have no known competing financial interests or personal relationships that could have appeared to influence the work reported in this paper.

## Data Availability

The authors do not have permission to share data.

## References

[bib1] Adamu A., Li S., Gao F., Xue G. (2024). The role of neuroinflammation in neurodegenerative diseases: current understanding and future therapeutic targets. Front. Aging Neurosci..

[bib2] Alshahrani S.M., Al-Kuraishy H.M., Albuhadily A.K. (2025). GDF15 upregulation in Parkinson's disease: compensatory mechanisms and causal insights. Cytokine.

[bib3] Amponsah J., Archibong-Eso A., Fiagbe Y.A.K. (2025). Interface-driven human brain injury mechanisms in blast exposure: a fluid–structure interaction model. Med. Nov. Technol. Dev..

[bib4] Appel-Cresswell S., de la Fuente-Fernandez R., Galley S., McKeown M.J. (2010). Imaging of compensatory mechanisms in parkinson's disease. Curr. Opin. Neurol..

[bib5] Balleine B.W., O'Doherty J.P. (2010). Human and rodent homologies in action control: corticostriatal determinants of goal-directed and habitual action. Neuropsychopharmacology.

[bib7] Belding J.N., Englert R.M., Fitzmaurice S. (2021). Potential health and performance effects of high-level and low-level blast: a scoping review of two decades of research. Front. Neurol..

[bib6] Belding J.N., Englert R., Bonkowski J., Thomsen C.J. (2021). Occupational risk of low-level blast exposure and TBI-related medical diagnoses: a population-based epidemiological investigation (2005-2015). Int. J. Environ. Res. Publ. Health.

[bib9] Bezard E., Crossman A.R., Gross C.E., Brotchie J.M. (2001). Structures outside the basal ganglia may compensate for dopamine loss in the presymptomatic stages of parkinson's disease. FASEB J..

[bib8] Bezard E., Gross C.E. (1998). Compensatory mechanisms in experimental and human parkinsonism: towards a dynamic approach. Prog. Neurobiol..

[bib10] Blanchard E.B., Jones-Alexander J., Buckley T.C. (1996). Psychometric properties of the PTSD checklist (PCL). Behav. Res. Ther..

[bib11] Blesa J., Trigo-Damas I., Dileone M., Del Rey N.L., Hernandez L.F., Obeso J.A. (2017). Compensatory mechanisms in parkinson's disease: circuits adaptations and role in disease modification. Exp. Neurol..

[bib12] Brotchie J., Fitzer-Attas C. (2009). Mechanisms compensating for dopamine loss in early parkinson disease. Neurology.

[bib13] Buchsbaum M.S., Simmons A.N., DeCastro A., Farid N., Matthews S.C. (2015). Clusters of low (18)F-Fluorodeoxyglucose uptake voxels in combat veterans with traumatic brain injury and post-traumatic stress disorder. J. Neurotrauma.

[bib14] Buhle J.T., Silvers J.A., Wager T.D. (2014). Cognitive reappraisal of emotion: a meta-analysis of human neuroimaging studies. Cerebr. Cortex.

[bib15] Champagne A.A., Coverdale N.S., Ross A., Murray C., Vallee I., Cook D.J. (2021). Characterizing changes in network connectivity following chronic head trauma in special forces military personnel: a combined resting-fMRI and DTI study. Brain Inj..

[bib16] Cicerone K., Kalmar K. (1995). Persistent postconcussion syndrome: the structure of subjective complaints after mild traumatic brain injury. J. Head Trauma Rehabil..

[bib17] Cregg J.M., DePaul M.A., Filous A.R., Lang B.T., Tran A., Silver J. (2014). Functional regeneration beyond the glial scar. Exp. Neurol..

[bib18] Davenport N.D., Lim K.O., Armstrong M.T., Sponheim S.R. (2012). Diffuse and spatially variable white matter disruptions are associated with blast-related mild traumatic brain in jury. Neuroimage.

[bib19] Dretsch M.N., Williams K., Staver T. (2017). Evaluating the clinical utility of the Validity-10 for detecting amplified symptom reporting for patients with mild traumatic brain injury and comorbid psychological health conditions. Appl. Neuropsychol. Adult.

[bib20] Fischl B. (2012). FreeSurfer. Neuroimage.

[bib21] Gao Z., Zhu Q., Zhang Y. (2013). Reciprocal modulation between microglia and astrocyte in reactive gliosis following the CNS injury. Mol. Neurobiol..

[bib22] Gharios C., van Leent M.M.T., Chang H.L. (2024). Cortico-limbic interactions and carotid atherosclerotic burden during chronic stress exposure. Eur. Heart J..

[bib23] Graybiel A.M. (2004). Network-level neuroplasticity in cortico-basal ganglia pathways. Park. Relat. Disord..

[bib24] Haber S.N. (2016). Corticostriatal circuitry. Dialogues Clin. Neurosci..

[bib25] Huang S., Lu Y., Fang W., Huang Y., Li Q., Xu Z. (2025). Neurodegenerative diseases and neuroinflammation-induced apoptosis. Open Life Sci..

[bib26] Hulkower M.B., Poliak D.B., Rosenbaum S.B., Zimmerman M.E., Lipton M.L. (2013). A decade of DTI in traumatic brain injury: 10 years and 100 articles later. AJNR Am. J. Neuroradiol..

[bib27] Ivanov I., Fernandez C., Mitsis E.M., Dickstein D.L., Wong E., Tang C.Y., Simantov J., Bang C., Moshier E., Sano M., Elder G.A., Hazlett E.A. (2017). Blast exposure, white matter integrity, and cognitive function in Iraq and Afghanistan combat veterans. Front. Neurol..

[bib28] Jenkinson M., Beckmann C.F., Behrens T.E., Woolrich M.W., Smith S.M. (2012). Fsl. Neuroimage.

[bib29] Jeurissen B., Leemans A., Tournier J.D., Jones D.K., Sijbers J. (2013). Investigating the prevalence of complex fiber configurations in white matter tissue with diffusion magnetic resonance imaging. Hum. Brain Mapp..

[bib30] Kong X.Z., Postema M.C., Guadalupe T. (2022). Mapping brain asymmetry in health and disease through the ENIGMA consortium. Hum. Brain Mapp..

[bib31] Lippa S.M., Yeh P.H., Kennedy J.E. (2023). Lifetime blast exposure is not related to white matter integrity in service members and veterans with and without uncomplicated mild traumatic brain injury. Neurotrauma Rep..

[bib33] Meabon J.S., Huber B.R., Cross D.J., Richards T.L., Minoshima S., Pagulayan K.F. (2016). Repetitive blast exposure in mice and combat veterans causes persistent cerebellar dysfunction. Sci. Transl. Med..

[bib35] Miller S.T., Cooper C.F., Elsbernd P., Kerwin J., Mejia-Alvarez R., Willis A.M. (2021). Localizing clinical patterns of blast traumatic brain injury through computational modeling and simulation. Front. Neurol..

[bib36] Mink J.W. (1996). The basal ganglia: focused selection and inhibition of competing motor programs. Prog. Neurobiol..

[bib37] Mink J.W. (2018). Basal ganglia mechanisms in action selection, plasticity, and dystonia. Eur. J. Paediatr. Neurol..

[bib38] Modica L.C.M., Egnoto M.J., Statz J.K., Carr W., Ahlers S.T. (2021). Development of a blast exposure estimator from a department of defense-wide survey study on military service members. J. Neurotrauma.

[bib39] Molina M.E., Isoardi R., Prado M.N., Bentolila S. (2010). Basal cerebral glucose distribution in long-term post-traumatic stress disorder. World J. Biol. Psychiatr..

[bib40] Ochsner K.N., Silvers J.A., Buhle J.T. (2012). Functional imaging studies of emotion regulation: a synthetic review and evolving model of the cognitive control of emotion. Ann. N. Y. Acad. Sci..

[bib42] Peskind E.R., Petrie E.C., Cross D.J. (2011). Neuroimage.

[bib43] Qian S., Ba Y., Xue L. (2025). Age-related changes of human brain metabolism. Eur. J. Nucl. Med. Mol. Imag..

[bib44] R Core Team (2021). https://www.R-project.org/.

[bib45] Roth R.H., Ding J.B. (2024). Cortico-basal ganglia plasticity in motor learning. Neuron.

[bib46] Smith J.A., Das A., Ray S.K., Banik N.L. (2012). Role of pro-inflammatory cytokines released from microglia in neurodegenerative diseases. Brain Res. Bull..

[bib47] Song C., Yeh P.H., Ollinger J. (2022). Altered metabolic interrelationships in the cortico-limbic circuitry in military service members with persistent post-traumatic stress disorder symptoms following mild traumatic brain injury. Brain Connect..

[bib48] Sours Rhodes C., Sandlain R., Ollinger J. (2023). Development of the blast ordnance and occupational exposure measure for self-reported lifetime blast exposures. Mil. Med..

[bib49] Stone J.R., Avants B.B., Tustison N.J. (2020). Functional and structural neuroimaging correlates of repetitive low-level blast exposure in career breachers. J. Neurotrauma.

[bib50] Stone J.R., Avants B.B., Tustison N.J., Gill J., Wilde E.A., Neumann K.D., Gladney L.A., Kilgore M.O., Bowling F., Wilson C.M., Detro J.F., Belanger H.G., Deary K., Linsenbardt H., Ahlers S.T. (2024). Neurological effects of repeated blast exposure in special operations personnel. J. Neurotrauma.

[bib51] Su E., Bell M., Laskowitz D., Grant G. (2016). Translational Research in Traumatic Brain Injury.

[bib52] Terry G., Pagulayan K.F., Muzi M. (2024). Increased [18F]Fluorodeoxyglucose uptake in the left pallidum in military veterans with blast-related mild traumatic brain injury: potential as an imaging biomarker and mediation with executive dysfunction and cognitive impairment. J. Neurotrauma.

[bib53] Wang H., Song G., Chuang H. (2018). Portrait of glial scar in neurological diseases. Int. J. Immunopathol. Pharmacol..

[bib54] Yuan W., Barber Foss K.D., Dudley J. (2019). Impact of low-level blast exposure on brain function after a one-day tactile training and the ameliorating effect of a jugular vein compression neck collar device. J. Neurotrauma.

[bib55] Zhang W., Xiao D., Mao Q., Xia H. (2023). Role of neuroinflammation in neurodegeneration development. Signal Transduct. Targeted Ther..

